# Temperament in infancy and behavioral and emotional problems at age 5.5: The EDEN mother-child cohort

**DOI:** 10.1371/journal.pone.0171971

**Published:** 2017-02-15

**Authors:** Xian Abulizi, Laura Pryor, Grégory Michel, Maria Melchior, Judith van der Waerden, on behalf of The EDEN Mother–Child Cohort Study Group

**Affiliations:** 1 Social Epidemiology Research Group, Institut Pierre Louis d’Épidémiologie et de Santé Publique (IPLESP), INSERM UMR_S 1136 & Sorbonne Universités- Pierre et Marie Curie, Paris, France; 2 INSERM Research Center for Epidemiology and Biostatistics (U897), Faculty of psychology, University of Bordeaux, Bordeaux, France; Royal Children's Hospital, AUSTRALIA

## Abstract

**Objective:**

Early temperamental characteristics may influence children’s developmental pathways and predict future psychopathology. However, the environmental context may also shape or interact with infant temperament and indirectly contribute to increased vulnerability to adverse developmental outcomes. The aim of the present study is to explore the long-term contribution of temperamental traits at twelve months of age to the presence of emotional and behavioral problems later in childhood, and whether this association varies with the child’s sex, parental separation, family socioeconomic status and maternal depression.

**Method:**

1184 mother-child pairs from the EDEN mother-child birth cohort study based in France (2003–2011), were followed from 24–28 weeks of pregnancy to the child’s fifth birthday. Infant temperament at 12 months was assessed with the Emotionality Activity and Sociability (EAS) questionnaire and behavior at 5.5 years was assessed with the Strengths and Difficulties Questionnaire (SDQ).

**Results:**

Emotional temperament in infancy predicts children’s overall behavioral scores (β = 1.16, p<0.001), emotional difficulties (β = 0.30, p<0.001), conduct problems (β = 0.51, p<0.001) and symptoms of hyperactivity/inattention (β = 0.31, p = 0.01) at 5.5 years. Infants’ active temperament predicts later conduct problems (β = 0.30, p = 0.02), while shyness predicts later emotional problems (β = 0.22, p = 0.04). The association between the child’s temperament in infancy and later behavior did not vary with children’s own or family characteristics.

**Conclusion:**

An emotional temperament in infancy is associated with higher levels of emotional and behavioral difficulties at the age of 5.5 years. Children who show high emotionality early on may require early prevention and intervention efforts to divert possible adverse developmental pathways.

## Introduction

Temperament involves constitutionally based individual differences in reactivity and self-regulation, in the domains of affect, activity and attention [1, 2]. Although a precise definition continues to be a subject of debate, the general consensus is that children’s temperamental traits manifest themselves early in life, have a strong genetic or neurobiological basis, and are relatively stable across time and situations [3, 4]. Research (to date) has mainly focused on the following dimensions of temperament: a) in infancy and early childhood: positive emotionality, activity level, fearfulness, anger/frustration, sociability, attentional orienting; b) in early childhood: effortful control, i.e. the capacity to inhibit a dominant response in order to perform a subdominant response [5, 6]. Infants who are prone to negative emotional expressions, low adaptability, high reactivity, and low emotional regulation have in the past been described as having a ‘‘difficult” temperament style [1, 7]. However, as the concept of difficult temperament includes a diverse set of characteristics that vary not only across studies but also across socio-cultural contexts [8], more recent studies advocate for an operational definition of temperament based on a number of specific dimensions or traits rather than the “difficult temperament” typology/classification [9].

Starting from infancy and toddlerhood, early temperamental characteristics may influence children’s developmental pathways and predict psychopathology later in childhood or adolescence [10, 11]. Several studies have identified that certain temperamental dimensions in infancy appear to predispose a child to develop specific forms of behavioral and emotional problems [12–14]. Particularly, negative emotional reactivity appears to be a non-specific risk factor for both internalizing and externalizing problems [5, 15]. High shyness and fearfulness have been found to specifically predict internalizing problems, particularly anxiety [16, 17]. High activity and lack of self-regulation predict externalizing problems [1, 18] and have been found to be related to attention-deficit hyperactivity disorder [19]. On the other hand, high sociability may function as a protective factor for behavior problem development, allowing a child to derive support from other sources in stressful situations [20].

The link between infant temperamental dimensions and later behavior problems is complex, with several mechanisms that may explain a longitudinal association. First, if temperament reflects a stable biological characteristic, this characteristic may manifest itself in the form of behavioral difficulties as the child grows older [15]. Twin and adoption studies indicate that genes influence individual differences in temperament, continuity and change in temperament and, at least in part, mediate the link between temperament and behavior problems [21, 22]. Further, neurobiological research has shown that specific areas of the prefrontal cortex and limbic structures are key regions associated with commonalities between temperamental dimensions and psychopathology [23]. Yet, despite a biological or even genetic basis, infant temperament does not necessarily predispose a child to later behavior problems. Its expression and impact on later emotional or behavior problems may be influenced by environmental or contextual factors [2, 20, 24]. This environmental context may directly contribute to increased vulnerability to adverse developmental outcomes, but also indirectly through its interaction with infant temperament [25]. The differential susceptibility hypothesis [26] posits that individuals with different temperamental characteristics will show different susceptibility to contextual factors. In recent years, this has been supported in particular by studies examining interactions between temperament and parenting on child maladjustment [1, 27]. However, little is known about interactions between infant temperament and other contextual risk factors in creating vulnerability for emotional and behavioral problems in early childhood.

Generally, risk factors influencing children’s psycho-social development can be divided into three categories: risks specific to the child (e.g. child sex), risks in the family and social context (e.g. parental marital problems, low socioeconomic status and poverty), and risks specific to parents (e.g. parental mental health) [13]. Gender differences exist in temperamental expression, with boys tending to have higher activity levels, while girls show higher effortful control [28]. However, studies showing how child gender might moderate relations between early temperament and later child behavior problems have been inconclusive. It has been suggested that boys follow specific temperamental trajectories [29], with high emotionality found to be associated with attention problems in boys [30], while others indicate that temperamental development may not differ between girls and boys [31]. The family and social environment have the potential to influence changes in children’s temperament. Parental separation and single parenthood have been found to be directly associated with children’s temperament, ability for emotion regulation and wellbeing [32]. Parents’ marital difficulties could pose a risk for children’s development [33], with some studies finding support for the idea that children who have an especially difficult temperament are at risk of adjustment problems when faced with low marital adjustment [34]. Concerning socioeconomic position, some studies find no or minimal evidence of a socioeconomic gradient in infant temperament [31], while in others an unambiguous socioeconomic gradient exists across various temperamental dimensions [35, 36]. Generally, children from disadvantaged families or living in less affluent neighborhoods are over-represented at the 'problematic' end of temperament dimensions, especially those relating to a difficult temperament [37, 38]. Using data from the EDEN mother-child cohort study, we previously reported that infants growing up with depressed mothers are more likely to have an emotional temperament characterized by high levels of negative reactivity, particularly if their family experiences socioeconomic difficulties [36]. While few studies have explicitly examined interactions between parental depression and child temperament, it appears that infant negative emotionality in particular moderates the association between maternal depressive symptoms and child behavior problems [39, 40].

Overall, outcomes from previous work on the interaction between infant temperament and contextual risks in influencing vulnerability to subsequent emotional and behavioral problems remain inconclusive and, additionally, have often focused on a global difficult temperament construct [25, 27]. To our knowledge, few studies have simultaneously examined the direct association between temperament dimensions and child maladjustment in early childhood as well as the moderating role of contextual risks other than parenting in this association. Greater understanding of potential moderating factors for child psychiatric outcomes may contribute to identifying at risk populations and aligning psychosocial research with effective and targeted prevention. Thus, the aim of the present study is to explore the long-term contribution of different temperamental traits at twelve months of age to the presence of emotional and behavioral problems later in childhood. As significant heterogeneity may exist in the relationship between early temperament and later behavioral or emotional problems, a second purpose of the study is to examine interactions between infant temperament dimensions and contextual factors (the child’s sex, parental separation and family socioeconomic status, maternal depression) in the development of child emotional and behavioral problems. We hypothesized that infants scoring high on the temperamental dimensions of emotionality, activity and shyness or low on temperamental sociability and experiencing adverse environmental conditions would be more susceptible to emotional or behavioral difficulties in the preschool period.

## Methods

### Study design and sample

The EDEN (Etude sur les déterminants pré et post natals précoces du Développement psychomoteur et de la santé de l’ENfant) mother-child cohort was set up to assess the pre- and postnatal nutritional, social, and environmental determinants of infant and child development and health [41]. Pregnant women were recruited before 24 weeks of gestation from two maternity wards (Poitiers and Nancy University hospitals in France) between September 2003 and January 2006. Exclusion criteria were multiple pregnancies, a known history of diabetes, the inability to speak and read French or plans to move out of the study region in the following 3 years. Among eligible women, 53% (n = 2002) agreed to participate and birth data were obtained from 1899 mother-infant pairs. During pregnancy and after birth (4, 8, 12, 24 months, 3, 4 and 5 years), socio-demographic and biomedical data on the mother and child were gathered a) from medical records, b) in face to face interviews with the mother and c) by mother’s self-completed questionnaires. At the 5-year follow-up, data were available for 1255 (66.0%) participating mothers and children. Attrition from the cohort was highest in young mothers (p < .001), those with low educational level (p < .001), of non-French origin (p < .001), who did not live with the father of their child (p = .002), as well as those who were depressed during pregnancy (p < .001) or in the postpartum period (p = .002). Written consent was obtained from the mother for herself at inclusion and for her newborn child after delivery. The EDEN study was approved by the Comité Consultatif de Protection des Personnes pour la Recherche Biomédicale (CPP, Ethics Committee, Kremlin Bicêtre Hospital) and by the Commission Nationale de l’Informatique et des Libertés (National Committee for Processed Data and Freedom (CNIL).

### Measures

#### Infant temperament

The Emotionality Activity Sociability (EAS) Temperament Questionnaire [42] was completed by mothers when the study child was 12 months old. Buss and Plomin propose four temperamental dimensions: emotionality (tendency to show distress, e.g. cries easily, reacts intensely when upset), activity (preferred level of activity, e.g. is always on the go, is very energetic), shyness (tendency to be inhibited with unfamiliar people, e.g. tends to be shy, takes a long time to warm up to strangers) and sociability (tendency to prefer the company of others, e.g. likes to be with people, prefers playing with others to playing alone). Some of these are conceptually similar to traits in other scales. For example, the Revised Infant Behaviour Questionnaire [43] includes dimensions of Extraversion/Surgency and Negative Affectivity, similar to EAS Activity/Sociability and Emotionality, respectively [44]. The EAS instrument consist of 20 items rated between 1 (my child’s behavior is never like this) and 5 (my child’s behavior is always like this). The scores from the questions belonging to each temperament are summed to form the four temperament dimensions. The EAS has satisfactory psychometric properties in children and infants and has been validated in France [45]. In the current sample, reliability was satisfactory with moderate-to-high internal consistency (Cronbach alphas = 0.63−0.72) for the different dimensions.

#### Child emotional and behavioral problems

The Strengths and Difficulties Questionnaire (SDQ), a well-known, well-validated and widely used screening questionnaire for behavior in of 3- to 16-year-olds [46, 47], was completed by mothers when their child was aged 5.5 years. The SDQ consists of 25 items that are divided into 5 subscales (range 0–10): emotional symptoms, conduct problems, symptoms of hyperactivity/inattention, peer relationship problems and pro-social behavior. The first four subscales are summed to obtain a score of children's overall behavior (range 0 to 40). In the present study, we focus on children’s overall behavior (a = 0.79 in the current sample) and the four subscales which are used to assess it.

#### Environmental risks

To determine the possible interaction of the environmental context with infant temperament, we included the following child or family characteristics assessed at the 12-months follow-up unless indicated otherwise: Child sex (male vs. female), family situation (parents living together vs. separated), family socioeconomic status (average household income in Euros per month, ordered categorically: 1: <800, 2: 800–1500, 3: 1500–2300, 4: 2300–3000, 5: 3000–3800, 6: >3800). Maternal depressive symptoms were assessed using the Edinburgh Postnatal Depression Scale (EPDS (Cox et al., 1987) at 12 months postpartum. Reliability for the EPDS was good, with a Cronbach alpha of 0.86.

#### Covariates

Multivariate analyses were adjusted for the following child, maternal and family characteristics ascertained at study baseline and significantly associated (p < .10) with temperament and child emotional and behavioral problems. *Child* characteristics included: premature birth (<37 vs. ≥37 weeks of gestation), birth weight (in grams), birth order (firstborn, no vs. yes). *Maternal* characteristics included: age at delivery, history of mental health problems (no vs. yes), depression in pregnancy (Center for Epidemiological Studies Depression (CES-D) questionnaire (Radloff, 1977), and anxiety in pregnancy (State-Trait Anxiety Inventory (STAI)[48]). *Family* characteristics were: study center (Poitiers vs. Nancy), parental education (mean maternal and paternal school years), paternal substance abuse (no vs. yes), self-reported social support (no vs. yes).

### Statistical analysis

Our aim was to test the association between children’s temperament at 12 months and their behavior at 5.5 years, and whether these were moderated by environmental risks. Analyses were based on participating children with complete data on the SDQ scale at the 5.5-year follow-up (n = 1184).

First, we began by computing descriptive statistics and bivariate associations between temperament, behavior and environmental risks were explored using Pearson correlation coefficients. Second, we tested associations between each of the EAS subscale scores (emotionality, activity, shyness, and sociability) and children’s behavior. After performing these univariate analyses, we included child sex, family socioeconomic status, parental separation and maternal depression in subsequent models.

Third, associations between trajectories of maternal depression and children’s behavior were studied controlling for previously listed covariates. Finally, we used the standard method of determining whether a moderating effect exists, which entails the addition of an interaction term (linear) between the four EAS subscales and the environmental risks in the multivariate regression models to test their independent associations with each child outcome. For all interactions, predictor variables were centered by subtracting their means to minimize multi-collinearity, and interaction terms were computed by multiplying predictor variables. In case of a significant interaction term, we conducted post-hoc probing using estimation of simple slopes [49] at 1 *SD* above and 1 *SD* below the sample mean for dichotomous moderators and by applying the Johnson-Neyman technique for continuous moderators [50]. All moderation analyses were run within the PROCESS package [51]. Missing data were imputed using multiple imputations by fully conditional specification. Excluding individuals with missing data from our analyses did not alter the significance of our results. All analyses were performed in the linear regression framework, using the SAS 9.3 statistical software.

## Results

### Sample descriptives

**Table 1** presents child, mother and family characteristics of our sample. Mothers were on average 30 years old at the time of birth. 15.1% had a history on mental health problems, while 22.2% and 18.2% reported respectively symptoms of depression or anxiety during pregnancy. Children’s parents were mostly (97%) married or cohabitating, with an average monthly income between 2300–3000 euros. Depressive symptoms during the postpartum year were common, with 15.3% of women reporting scores that fell above the 11-point cut-off. For the whole population, mean behavioral and emotional scores were within the normal range, with no significant differences in infant temperamental scores between those who completed the SDQ at age 5 and those who did not. Bivariate correlations between temperament, behavior measures and the environmental risk factors are presented in **Table 2**. The four EAS scales showed significant correlations between them, as well as with the different child emotional and behavioral outcomes. The environmental risks generally showed small to moderate correlations with both infant temperament and child behavior problems.

**Table 1:** Child, mother and family characteristics of EDEN cohort study participants (n = 1903).

	*Completers SDQ (n = 1184)*	*Non completers SDQ (n = 719)*	*P value* ^*¥*^
***Child characteristics***			
Sex (male)	626 (52.9)	374 (52.0)	0.72
Preterm birth (<37 weeks)	68 (5.7)	42 (5.8)	0.94
Birth weight (grams)	3292.7 (515.7)	3255.4 (505.9)	0.12
Birth order (firstborn)	551 (46.5)	299 (41.5)	0.03
***Maternal characteristics***			
Age at delivery (years)	30.13 (4.70)	28.4 (5.02)	< .001
History of mental health problems (yes)	179 (15.1)	13 (6.8)	.001
Maternal anxiety (STAI) in pregnancy	10.04 (9.65)	11.96 (10.22)	< .001
Maternal depression (CESD) in pregnancy	10.95 (7.69)	12.94 (8.63)	< .001
Maternal depression (EPDS) 1 year	4.33 (4.51)	5.39 (5.17)	< .001
***Family characteristics***			
Study center (Poitiers)	555 (46.9)	479 (58.6)	< .001
Parental educational level (years)	13.66 (2.32)	12.68 (2.30)	< .001
Family situation			< .001
Separated	36 (3.0)	47 (8.3)	
Family income (1–6)	4.26 (1.26)	3.67 (1.53)	< .001
Paternal alcohol abuse (yes)	14 (1.2)	14 (2.1)	0.13
Social support (no)	326 (27.6)	267 (36.3)	< .001
***Children’s EAS scores at 12 months***			
Emotionality	2.78 (0.71)	2.83 (0.72)	0.08
Activity	3.53 (0.48)	3.54 (0.54)	0.52
Sociability	3.69 (0.61)	3.72 (0.63)	0.48
Shyness	2.09 (0.57)	2.10 (0.63)	0.64
***Children’s behavioral scores at age 5*.*5 (cut-point for scores at clinical level)***			
Emotional symptoms *(≥4)*	2.13 (1.88)	--	
Conduct problems *(≥5)*	2.36 (2.04)	--	
Peer relationship problems *(≥2)*	1.20 (1.32)	--	
Hyperactivity/inattention *(≥6)*	3.07 (2.39)	--	
Overall behavioral score *(≥14)*	8.75 (5.21)	--	

EAS: Emotionality Activity Sociability scale; SDQ: Strengths and Difficulties Questionnaire. No. (%) or mean (SD) for continuous variables.

^¥^ P value of ANOVA for quantitative variables and Χ2 for categorical variables.

**Table 2:** Bivariate correlations between temperament, behavioral scores and environmental risk factors (n = 1184).

	1	2	3	4	5	6	7	8	9	10	11	12	13
1. Emotionality	1.00												
2. Activity	.05*	1.00											
3. Sociability	.19**	.21**	1.00										
4. Shyness	.21**	-.23**	-.33**	1.00									
5. Emotional symptoms	.17**	-.01	.02	.08**	1.00								
6. Conduct problems	.20**	.10**	.05	.00	.20**	1.00							
7. Peer relationship symptoms	.05	-.07*	-.07*	.04	.30**	.25**	1.00						
8. Hyperactivity/inattention	.12**	.09**	.00	-.06*	.19**	.48**	.21	1.00					
9. Overall behavioral score	.21**	.05	.01	.01	.60**	.74**	.55**	.77**	1.00				
10. Child sex	.01	-.08**	.02	.10*	.01	-.14**	-.04	-.14**	-.13**	1.00			
11. Family status	.01	.03	-.01	.00	.03	.08**	.05	.05	.08**	.01	1.00		
12. Family income	-.13**	-.01	.06*	-.02	-.07*	-.11**	-.13**	-.23**	-.20**	.03	-.33**	1.00	
13. Maternal depression	.21**	-.02	-.02	.07*	.17**	.21**	.19**	.16**	.26**	.04	.05*	-.15**	1.00

p≤ 0.05 (*), 0.01 (**).

### Infant temperament and child behaviour

**Table 3** displays results of linear regression models testing associations between children's temperament in infancy and behavior at age 5.5 years. Overall, we observed statistically significant associations between infants’ emotionality levels and overall behavior in later childhood (β = 1.68, p<0.001). In the fully adjusted model, the association between emotionality in infancy and overall behavior problems at age 5.5 years decreased but remained positive and statistically significant (β = 1.16, p<0.001). In particular, an emotional temperament in infancy predicts emotional difficulties (β = 0.30, p<0.001), conduct problems (β = 0.51, p<0.001) and symptoms of hyperactivity/inattention (= 0.31, p<0.01). We found no evidence that other aspects of children’s early temperament were associated with later overall behavior, but children’s active temperament predicted later conduct problems (β = 0.30, p = 0.02) and fewer peer relationship problems (β = -0.19, p = 0.02) while shyness predicted later emotional problems (β = 0.22, p = 0.04) and fewer symptoms of hyperactivity/inattention (β = -0.31, p = 0.02).

**Table 3:** Temperament at 12 months and children’s behavioral scores at age 5.5 years in the EDEN cohort study stepwise linear regression models (n = 1184, 2003–2011 France, β, 95% CI, p-value).

Children’s behavioral scores										
	Emotional symptoms		Conduct problems		Peer relationship problems		Symptoms of hyperactivity/ inattention		Overall behavioral score	
	*B (95% CI)*	*P*	*B (95% CI)*	*P*	*B (95% CI)*	*p*	*B (95% CI)*	*p*	*B (95% CI)*	*p*
EAS dimension										
***Unadjusted model***										
Emotionality	.43 (.25 -.58)	.00***	.62 (.43- .79)	.00***	.14 (.02 -.26)	.02*	.52 (.31- .72)	.00***	1.68 (1.24–2.13)	.00***
Activity	-.08 (-.29 -.18)	.51	.33 (.08 -.59)	.01**	-.17 (-.33 -.00)	.06	.36 (.05- .64)	.02**	.46 (-.18–1.11)	.16
Sociability	.03 (-.14 -.26)	.77	-.08 (-.30- .14)	.48	-.16 (-.30–-.02)	.03*	-.28 (-.53–-.03)	.03*	-.49 (-1.03- .05)	.07
Shyness	.14 (-.04 -.39)	.20	-.10 (-.34 - .13)	.39	-.04 (-.19 -.12)	.63	-.40 (-.69–-.15)	.01**	-.41 (-.98- .17)	.17
*R*^*2*^	0.03		0.05		0.01		0.03		0.05	
**Adjusted model I**^a^										
Emotionality	.38 (.21- .55)	.00***	.52 (.34- .69)	.00***	.08 (-.04- .19)	.21	.38 (.17- .59)	.00***	1.35 (.91–1.79)	.00***
Activity	-.08 (-.32- .15)	.49	.27 (.02-.52)	.03*	-.18 (-.35–-.02)	.03*	.31 (.02- .60)	.03*	.34 (-.28- .96)	.29
Sociability	.04 (-.15- .24)	.66	.01 (-.22-.21)	.95	-.12 (-.26- .02)	.10	-.15 (-.39- .09)	.23	-.24 (-.77- .28)	.36
Shyness	.12 (-.09- .34)	.26	-.08 (-.31- .15)	.49	-.04 (-.19- .11)	.60	-.36 (-.63–-.10)	.01**	-.37 (-.93- .20)	.21
*R*^*2*^	0.05		0.10		0.05		0.10		0.13	
**Adjusted model I**^b^										
Emotionality	.30 (.13- .46)	.00***	.51 (.33- .69)	.00***	.04 (-.08- .16)	.49	.31 (.11- .52)	.01**	1.16 (.72–1.60)	.00***
Activity	-.10 (-.33- .13)	.40	.30 (.05- .55)	.02*	-.19 (-.36–-.03)	.02*	.27 (-.01- .56)	.06	.28 (-.34- .90)	.37
Sociability	.11 (-.08- .31)	.26	.01 (-.18- .25)	.93	-.07 (-.21- .07)	.31	-.04 (-.29- .20)	.72	.10 (-.53–50)	.96
Shyness	.22 (.01- .43)	.04*	-.12 (-.22- .20)	.32	-.02 (-.17- .13)	.81	-.31 (-.58–-.05)	.02*	-.23 (-.79- .34)	.43
*R*^*2*^	0.11		0.12		0.08		0.15		0.17	

p≤ 0.05 (*), 0.01 (**), 0.001 (***)

^a^ adjusted for child’s sex, parental separation, family income and maternal depression

^b^ adjusted for model I^a^ and study center, premature birth, birthweight, birth order, maternal age at birth, maternal history of mental health problems, maternal anxiety and depression in pregnancy, social support, parental educational level, paternal substance abuse.

### Moderator analyses

First, we observed main effects of child sex, with girls having fewer overall behavioral problems compared to boys, as well as maternal depression being associated with child problem behavior in the expected direction. When entering in the regression models the interaction terms between the EAS temperamental dimensions and the proposed environmental risks, associations fell short of statistical significance (**Table 4).** The association between the child’s emotional temperament in infancy and later behavior did not vary with gender (β = -0.38; 95% CI [(-1.21–0.45], p = 0.37), family socioeconomic status (β = -0.20; 95% CI [-0.55–0.15], p = 0.26), parental separation (β = 1.68; 95% CI [-1.65–5.00], p = 0.32) or maternal depression (β = -0.02; 95% CI [-0.11–0.06], p = 0.58). Nor did the activity, sociability and shyness temperament scores have significant interactions with the proposed moderators (**S1 Table**).

**Table 4:** Temperament at 12 months and children’s overall behavioral score at age 5.5 years in the EDEN cohort study- moderation analyses.

	Children’s Overall Behavioral Score	
Predictors	*B (95% CI)*	*p*
Emotionality	1.16 (.72–1.60)	.001*
Activity	.28 (-.34- .90)	.37
Sociability	-.01 (-.54- .51)	.96
Shyness	-.23 (-.79- .34)	.43
Child sex (female)	-1.60 (-2.16–-1.01)	.001*
Family status (single)	1.15 (-.72–3.02)	.23
Family income	-.12 (-.42- .18)	.42
Maternal depression	.19 (.12- .26)	.001*
**Interactions**		
***Child sex***		
Emotionality *sex	-.38 (-1.21- .45)	.37
Activity * sex	.38 (-.84–1.60)	.54
Sociability * sex	.95 (-.02–1.92)	.05
Shyness * sex	-.62 (-1.65- .41)	.24
***Family status***		
Emotionality * Family status	1.68 (-1.65–5.00)	.32
Activity * Family status	-2.95 (-6.78- .88)	.13
Sociability * Family status	-2.23 (-5.25- .78)	.15
Shyness * Family status	1.41 (-1.20–4.03)	.29
***Family income***		
Emotionality * Family income	-.20 (-.55- .15)	.26
Activity * Family income	-.01 (-.47- .46)	.98
Sociability * Family income	-.21 (-.60- .18)	.29
Shyness * Family income	-.01 (-.41- .38)	.95
***Maternal depression***		
Emotionality * Maternal depression	-.02 (-.11–06)	.58
Activity * Maternal depression	.06 (-.08 - .20)	.37
Sociability* Maternal depression	.01 (-.09 - .10)	.89
Shyness * Maternal depression	.02 (-.09 - .12)	.74

Adjusted for study center, premature birth, birth weight, birth order, maternal age at birth, maternal history of mental health problems, maternal anxiety and depression in pregnancy, social support, parental educational level, paternal substance abuse

p≤ 0.001 (*)

## Discussion

### Main findings

The objectives of this research were to determine the contribution of different temperamental traits at twelve months of age to the presence of emotional and behavioral problems later in childhood and whether children’s own or their families’ characteristics moderate this association. We found that in particular an emotional temperament in infancy predicts higher levels of emotional and behavioral problems scores at age 5.5 years. Moreover, an active temperament in infancy was associated with conduct problems at age 5.5 years, while shyness was associated with emotional problems. However, contrary to expectations, these associations do not appear to vary with children’s sex or family socioeconomic status, parental separation or maternal depression.

### Infant temperament and child behavior

Our results indicate that, even after controlling for a broad range of confounders, early temperamental traits predicted children’s emotional or behavioral difficulties at preschool age. Like the present study, Sayal et all [24] also observed that infant temperament measured at 6 months of age is associated with the presence of a psychiatric disorder at the age of 7 years. In particular, our data indicate continuity between early temperamental traits and subsequent problems in domains of internalizing and externalizing difficulties. A highly active temperament in infancy predicted later conduct problems. Previous studies have likewise reported that low effortful control and high activity are temperamental characteristics associated with childhood oppositional behavior, conduct problems, and ADHD [1, 10]. Further, shyness was associated with later emotional symptoms. A recent meta-analysis reported that inhibited temperament during infancy may persist across early development, and predict inhibited behaviors in early childhood [52]. Importantly, these childhood differences appear to predict long-term psychiatric outcomes, with inhibited children showing a fourfold increase in risk for developing social anxiety disorder [53]. Some evidence also suggests that a high EAS shyness score measured in early childhood may be associated with later depression [54]. Only high emotionality was associated with both emotional and conduct problems in our study. It has previously been reported that an emotional temperament is one of be the strongest predictors of later anxious or depressed behavior and attention problems in early childhood up to 7 years of age [30]. This indicates that this trait is a non-specific predictor of a wide variety of internalizing and externalizing difficulties, which is consistent with previous reports [5].

The direct associations between temperamental traits and later behavioral/emotional problems corresponded with what has been found in several past studies. However, with the exception of emotional temperament, it is important to note that associations remain weak, especially compared to certain other predictors. The link between infant temperament and later behavior problems is complex and probably involves different mechanisms. It has been argued that temperament represents the early substrate of childhood and adult psychiatric disorders, partly reflecting epigenetic influences. Analyses of temperament dimensions as possible endophenotypes for clinical disorders may, therefore, be fruitful [4]. Constitutional factors or gene-environment interactions could also, to some extent account for temperament- behavior continuities [21, 22]. Further, temperament and behavior problems could share common antecedents. For instance, maternal stress (including anxiety and depression) during pregnancy is associated with both temperament and behavioral problems in offspring [3, 55]. However, we controlled for these prenatal risks in our analyses. Others have proposed that temperament impacts child outcomes in an interactive person-environment fashion. To account for environmental effects in models of temperament and psychopathology, most previous research controlled for family socioeconomic status. We have extended this by taking into account other family and ecological risks that have been found to exert negative impact on child temperament as well as the emergence of child problem behavior. Thus the consequences of temperamental traits in infancy may have been obscured by other environmental characteristics that can contribute to the development of adjustment problems, including factors in the child, the family and the social context [6, 25, 56]. Male sex, being the first-born child, maternal depression, as well as low parental educational level and lack of social support were associated with overall worse behavioral scores at 5.5 years. Although boys and girls seem to display different patterns of behavior problems during childhood, reliable sex differences in both temperament and psychopathology do not emerge until the late preschool period [57, 58]. Low family socioeconomic status is associated with high levels of temperamental and behavioral difficulties in children [35, 56]. It has been suggested that one underlying pathway may be through parental support; higher SES families may be more knowledgeable about child development and more likely to have resources to buffer stress, thus protecting their children [31]. Finally, temperament and emotion regulation have also been found to affect peer interactions, and jointly predict numerous aspects of social competence, including social skills, prosocial behavior, adjustment, and peer acceptance [59]. Young children who consistently have problems managing emotions such as anger and distress tend to be at greater risk of poor social functioning, externalizing behavior problems, difficulties with peers and later psychopathology [60].

### Interactions of infant temperament with contextual risks

Our results extend the limited number of studies looking beyond simple linear relationships between a particular temperament trait and vulnerability to psychopathology and investigating how infant temperament might interact with other risk factors. In the present study, we hypothesized that infants scoring high on certain temperamental dimensions and experiencing adverse environmental conditions such as low family socio-economic status, parental separation or maternal depression would be more susceptible to have emotional or behavioral difficulties in the preschool period. However, contrary to our expectations, we observed no notable interaction between temperament and these contextual risks. Previously, an Australian longitudinal cohort equally showed that the very early experience of a moderate level of life stress within the family environment did not substantially interact with the temperament styles [25].Yet, other characteristics of children’s psychosocial environment could yield inter-individual differences in the consequences of temperamental traits [2, 11]. One factor that has extensively been studied in this context is parenting, as parent-child interactions may contribute to children’s problems [61, 62]. Children with tendencies toward negative emotionality and poor self-regulation can be challenging to handle and elicit more negative responses (e.g. less supportive parenting and more restrictive control) from their parents than their “easy” counterparts [37]. Experiences of parenting may in turn promote stability among children with these temperament profiles [63], which could contribute to higher levels of vulnerability to behavioral and psychiatric difficulties in the long run. Interestingly, there is no consensus in the literature regarding the direction of this effect; it is equally possible that the child’s temperamental characteristics may actually drive parenting behaviors [9]. In this light, it is possible that some of our proposed moderators might actually impact child behavior through more complex pathways of moderated mediation or conditional processes. For instance, maternal depression has consistently been found to have detrimental consequences on children’s emotional and behavioral development [64]. Thus, it is likely that the stressful family environment created by maternal depression and compromised parenting behaviors of depressed mothers influence the child’s temperamental traits and directly contribute to children’s later emotional and behavioral problems [39, 65]. Preliminary support for these mechanisms have been found by Dix and Yan [66] who reported that as mothers’ depressive symptoms increase, children who were high in negative emotionality at 6 months were particularly likely to be exposed to negative parenting, and negative parenting, in turn, predicted a range of problematic indices of adjustment when children were 3 years old. Future studies that examine specific genetic and environmental mechanisms of risk and resiliency will be useful to test these hypotheses in further detail.

### Strengths and limitations

Our research has several strengths. First, it is based on a large sample of families drawn from the general population. Second, this sample was followed prospectively. Third, several covariates such as parental separation, financial difficulties and maternal depression were assessed longitudinally. Fourth, infant temperament at 12 months and child behavior at 5.5 years were measured by internationally validated scales. Nevertheless, we also acknowledge limitations. First, families who had financial difficulties and less educated mothers were more likely to withdraw from participation before the 5.5 years follow-up. This selective attrition could reduce the generalizability of our findings, but is unlikely to have influenced the association between early emotionality and behavior later in childhood, which is our main finding. However, our outcomes relating to significant moderators could be conservative. Further, compared with a national perinatal survey performed in 2003 on a representative sample of French women, the EDEN study included at study inclusion a larger proportion of women with university level education (53 vs. 43%) [41]. Finally, although estimates of depression in the prenatal and postpartum period were elevated (22.0% and 15.3% respectively), this figure corresponds to prevalence rates reported both in both France and in other countries [67, 68]. Second, as in many other studies, mothers were the sole reporters of the child’s temperament and behavior problems. Thus, maternal characteristics (e.g. depression) might have influenced assessment of children’s temperament and later behavior in a negative way [69, 70]. Reassuringly, research suggests that maternal reports of children’s behavior tend to be valid even when the mother is depressed, therefore this methodological characteristic should not result in significant information bias [71]. Still, assessments from different informants (e.g. the child’s father, the child him/herself, teachers) may yield more valid and precise measures of children’s behavior than maternal reports only and should be favored in future research designs [72]. Finally, we were not able to examine the role of paternal psychopathology, which was not measured in the EDEN study. However, we accounted for paternal alcohol abuse, which is often associated with other mental health difficulties, thereby probably partly capturing the variability associated with paternal psychopathology [73].

### Implications

Our findings have potential implications for early prevention of emotional and behavioral difficulties in children. Investments should target the development of independent and tailored preventive interventions aimed at addressing both temperamental and contextual risks. While factors such as child sex or family characteristics are not modifiable, maternal depression has a direct impact on the child and may be reduced through appropriate interventions. A recent review reported that psychosocial and psychological interventions, compared to usual postpartum care, were associated with a reduction in depressive symptomatology within the first 12 months postpartum [74]. Additionally, research shows that interventions helping to decrease maternal stress in the perinatal period (e.g. the practice of mindfulness during pregnancy) can have positive effects on children’s temperament (lower levels of self-regulatory problems and “difficult” temperament at age 10 months [75]); in the long-run such interventions could also reduce levels of emotional and behavioral disturbances in children.

Temperamental traits in infancy, though modestly predictive of later behavior problems, generally reflect variations within the normal range. However, some studies have noted that parental perceptions of difficult infant temperament doubled a child’s risk for preschool problems, irrespective of the actual temperament category [56]. Chess and Thomas [7] consider these parent-child interactions within a goodness-of-fit framework, wherein optimal development can be achieved when there is a match the child’s temperament to the demands and expectations of the environment. Conversely, poorness of fit leads to maladaptive functioning. Thus, promoting supportive parent–infant interactions may be another way to buffer the long-term consequences of early emotionality. For example, an intervention proposing targeted behavior management recommendations familiarized parents with temperament-related behaviors that are likely to occur and are “normal” for their child [76].

### Conclusion

In conclusion, while more extreme levels of particular temperamental traits in infancy may not be problematic in and of themselves, an emotional temperament in particular can be a precursor to certain types of emotional and behavioral problems later in childhood. Contextual risk factors, such as child sex, family socioeconomic status, parental separation and maternal depression did not moderate this association, but were to some extent associated with later problem behavior. However, as these contextual risks are often not modifiable, primary health care professionals should be aware that children who show high emotional reactivity early on may require additional attention and follow-up to prevent emotional and behavioral difficulties later on. By providing support and education to parents, their children’s possible adverse developmental pathways may be diverted.

## Supporting information

S1 Table Temperament at 12 months and children’s behavioral scores at age 5.5 years in the EDEN cohort study- moderation analyses. Adjusted for study center, premature birth, birth weight, birth order, maternal age at birth, maternal history of mental health problems, maternal anxiety in pregnancy, maternal depression in pregnancy, social support, paternal substance abuse.
(DOCX)
