# Supplementary material for: Temperament in infancy and behavioral and emotional problems at age 5.5: The EDEN mother-child cohort
Source: PLoS One. 2017 Feb 15;12(2):e0171971. doi: 10.1371/journal.pone.0171971 (PMC5310866; doi:10.1371/journal.pone.0171971)
Supplement: S1 Table — Adjusted for study center, premature birth, birth weight, birth order, maternal age at birth, maternal history of mental health problems, maternal anxiety in pregnancy, maternal depression in pregnancy, social support, paternal substance abuse. (DOCX) [file pone.0171971.s001.docx]

|  | **Emotional symptoms** | | **Conduct problems** | | **Peer relationship problems** | | | | **Symptoms of hyperactivity/ inattention** | | |  |
| --- | --- | --- | --- | --- | --- | --- | --- | --- | --- | --- | --- | --- |
| **Predictors** | ***Β (95% CI)*** | ***p*** | ***Β (95% CI)*** | ***p*** | | ***Β (95% CI)*** | | ***p*** | | ***Β (95% CI)*** | ***p*** |  |
| **Interactions** |  |  |  |  | |  | |  | |  |  |  |
| ***Child sex*** |  |  |  |  | |  | |  | |  |  |  |
| Emotionality*sex | 0.07 (-0.23-0.38) | 0.63 | -0.04 (-0.17- 0.09) | 0.52 | | -0.14 (-0.35-0.08) | | 0.22 | | -0.21 (-0.59- 0.16) | 0.26 |  |
| Activity *sex | 0.16 (-0.28-0.61) | 0.47 | 0.21 (-0.27-0.70) | 0.38 | | 0.19 (-0.13-0.52) | | 0.23 | | -0.25 (-0.80-0.30) | 0.37 |  |
| Sociability*sex | 0.25 (-0.10-0.61) | 0.16 | 0.23 (-0.16-0.61) | 0.25 | | 0.14 (-0.12-0.40) | | 0.29 | | 0.06 (-0.38-0.50) | 0.78 |  |
| Shyness*sex | 0.31 (0.11-0.50) | 0.94 | -0.33 (-0.74-0.08) | 0.11 | | 0.07 (-0.21-0.34) | | 0.62 | | -0.17 (-0.64- 0.30) | 0.48 |  |
| ***Family status*** |  |  |  |  | |  | |  | |  |  |  |
| Emotionality* Family status | 0.02 (-1.19-1.22) | 0.97 | 0.21 (-0.24-0.66) | 0.36 | | 0.19 (-0.10-0.49) | | 0.21 | | 0.86 (-0.66-2.39) | 0.27 |  |
| Activity * Family status | -0.86 (-2.26-0.55) | 0.23 | -1.11 (-2.76-0.53) | 0.19 | | -1.03 (-2.11-0.05) | | 0.06 | | -0.46 (-2.32-1.39) | 0.62 |  |
| Sociability* Family status | -0.22 (-1.32-0.89) | 0.70 | -1.09 (-2.34-0.16) | 0.09 | | -0.71 (-1.53 -0.11) | | 0.09 | | -0.23 (-1.64-1.18) | 0.75 |  |
| Shyness* Family status | 0.47 (-0.48-1.43) | 0.33 | 0.95 (-0.10-2.00) | 0.08 | | 0.48 (-0.21-1.17) | | 0.48 | | -0.53 (-1.72-0.66) | 0.38 |  |
| ***Family income*** |  |  |  |  | |  | |  | |  |  |  |
| Emotionality* Family income | -0.07 (-0.20-0.05) | 0.27 | 0.03 (-0.02-0.08) | 0.27 | | -0.08 (-0.17--0.01) | | 0.07 | | -0.04 (-0.20- 0.11) | 0.60 |  |
| Activity * Family income | 0.10 (-0.07-0.27) | 0.24 | -0.03 (-0.10-0.04) | 0.42 | | 0.02 (-0.02-0.07) | | 0.30 | | -0.09 (-0.30- 0.12) | 0.41 |  |
| Sociability* Family income | -0.06 (-0.20-0.08) | 0.42 | -0.05 (-0.11-0.01) | 0.08 | | -0.03 (-0.07-0.01) | | 0.09 | | -0.07 (-0.25-0.10) | 0.41 |  |
| Shyness* Family income | 0.04 (-0.10-0.19) | 0.56 | 0.02 (-0.04-0.08) | 0.50 | | -0.03 (-0.07- 0.01) | | 0.13 | | 0.00 (-0.17-0.18) | 0.98 |  |
| ***Maternal depression*** |  |  |  |  | |  | |  | |  |  |  |
| Emotionality* Maternal depression | -0.01 (-0.05-0.03) | 0.66 | -0.02 (-0.05-0.02) | 0.38 | | 0.01 (0.00-0.02) | | 0.24 | | -0.01 (-0.05-0.03) | 0.65 |  |
| Activity * Maternal depression | 0.01 (-0.05-0.07) | 0.77 | 0.03 (-0.03-0.08) | 0.36 | | | 0.03 (0.00-0.07) | 0.07 | 0.03 (-0.07-0.06) | | 0.99 | |
| Sociability* Maternal depression | 0.02 (-0.02-0.06) | 0.39 | -0.01 (-0.03-0.04) | 0.89 | | | 0.01 (0.00-0.02) | 0.06 | 0.00 (0.00-0.01) | | 0.64 | |
| Shyness* Maternal depression | 0.00 (-0.05-0.04) | 0.83 | 0.02 (-0.02-0.07) | 0.26 | | | 0.01 (0.00-0.02) | 0.11 | 0.00 (-0.01-0.01) | | 0.78 | |

**S1 Table- Temperament at 12 months and children’s behavioral scores at age 5.5 years in the EDEN cohort study- moderation analyses**

*Adjusted for study center, premature birth, birth weight, birth order, maternal age at birth, maternal history of mental health problems, maternal anxiety in pregnancy, maternal depression in pregnancy, social support, paternal substance abuse*
